# Inequalities in hepatocellular carcinoma (HCC) surveillance uptake in London: a UK multicentre cohort study

**DOI:** 10.1136/bmjph-2026-005067

**Published:** 2026-10-09

**Authors:** Maria Qurashi, Varsha Chaitanya Bhide, Shahid A Khan, Nina Stafford, Jayshri Shah, Luke D Tyson, Zinu Philipose, Hooshang Izadi, Rohini Sharma

**Affiliations:** 1 Department of Surgery & Cancer, Imperial College London, London, UK; 2 Department of Metabolism, Digestion and Reproduction, Imperial College London, London, UK; 3 Hepatology, London North West Healthcare NHS Trust, London, UK; 4 Department of Hepatology, Croydon University Hospitals NHS Trust, London, UK; 5 Department of Mechanical Engineering and Mathematical Sciences, Oxford Brookes University, Oxford, UK

**Keywords:** Population Surveillance, Sociodemographic Factors, Epidemiologic Factors

## Abstract

**Introduction:**

Hepatocellular carcinoma (HCC) is the third-leading cause of cancer-related mortality worldwide. 6 monthly ultrasound surveillance for HCC is recommended by professional bodies, but fewer than 25% of patients receive surveillance at recommended intervals. The aim of this study was to describe surveillance uptake across a multicentre cohort, examining sociodemographic factors and predictors of surveillance attendance.

**Methods:**

Electronic healthcare data from North West London primary care were used to identify numbers of patients with chronic liver disease between March 2021 and March 2023. A case review of patients eligible for HCC surveillance was then conducted using hospital care records. Data were collected including surveillance attendance and sociodemographic factors. Surveillance attendance was classified: regular (four surveillance episodes in 2 years), infrequent (less than four attendances) or non-attendance. Regression analysis was undertaken to examine factors associated with regular or reduced attendance.

**Results:**

2195 patients were identified as having cirrhosis from primary care; 869 patients were identified from hospital care records as being under the care of hepatology/gastroenterology clinics and eligible for surveillance. Of these, 17% patients received regular surveillance; 73% of patients were in receipt of infrequent surveillance and 10% received no surveillance over the study period. The strongest predictor of regular surveillance was care under a specialist hospital (OR 0.08, 95% CI 0.04 to 0.2, p<0.001). People who were not deprived were more likely to be regular attenders (OR 0.4, 95% CI 0.2 to 0.8, p=0.008).

**Conclusions:**

Rates of regular HCC surveillance are low, with patients under the care of non-specialist centres most at risk of reduced attendance at surveillance. Our findings highlight the urgent need to address low surveillance uptake.

**WHAT IS ALREADY KNOWN ON THIS TOPIC**

- Despite guidelines recommending biannual surveillance in patients at high risk of hepatocellular carcinoma (HCC), uptake of HCC surveillance is low. Meta-analyses showing surveillance uptake in line with recommendations is <10% globally. There are no UK multicentre studies examining surveillance uptake or its determinants in HCC.

**WHAT THIS STUDY ADDS**

- In this multicentre study assessing HCC surveillance uptake, only 17% of patients received the recommended biannual surveillance over a 2-year study period. Patients of a lower socioeconomic status and those under the care of a non-specialist hospital were less likely to receive regular surveillance.

**HOW THIS STUDY MIGHT AFFECT RESEARCH, PRACTICE OR POLICY**

- This study highlights the urgent need to improve surveillance uptake, particularly among patients experiencing deprivation.

## Introduction

 Hepatocellular carcinoma (HCC) is the third leading cause of cancer-related death worldwide and, unlike other cancer types, incidence and mortality rates continue to rise.^1^ However, prognosis remains dismal: despite recent advances in treatment options, 5-year survival rates are <20%.^2^ This is attributable to late-stage presentation with advanced disease, which is not amenable to curative treatment.

90% of HCC cases arise on a background of chronic liver disease (CLD) and its sequelae, cirrhosis.^2^ It is estimated that a third of patients with cirrhosis will develop HCC during their life-span, with a reported annual incidence rate of 1–8%.^2^ There exists, therefore, a clearly identifiable target ‘at risk’ population. Surveillance for HCC involves 6 monthly liver ultrasound (US)±alpha-fetoprotein and is recommended by all international professional bodies,^2,3^ with the aim of early cancer detection and improved HCC-related mortality. The landmark study evaluating HCC surveillance found that US surveillance for patients with hepatitis B (HBV)-related cirrhosis reduced mortality from HCC by 37% when compared with no surveillance.^4^ Following this study, there have been numerous cohort and modelling studies assessing the utility of HCC surveillance: a recent meta-analysis of these has demonstrated that biannual surveillance improves early cancer detection, receipt of curative treatment and survival.^5,6^

Wolf and colleagues published a meta-analysis of global surveillance uptake comprising 29 studies conducted after 2010, with a total of nearly 1 20 000 patients.^7^ The majority (66%) of studies were from North America, with the remainder from Europe (24%), Asia (7%) and South America (3%). There were no UK studies in this meta-analysis. The pooled estimate of surveillance uptake among eligible patients was 24%, although with heterogeneity across studies. Subgroup analyses found that surveillance receipt was significantly lower in the USA (17.8%) compared with Europe (43.2%) and Asia (34.6%). Additionally, surveillance was highest in studies evaluating patients receiving subspecialist gastroenterology or hepatology care (77%), compared with population-based cohort studies (8%).

Barriers to HCC surveillance have been identified in studies from the USA, and are thought to be multifactorial, with patient, clinician and system factors identified.^8^ A population-based cohort study of HCC cases across the USA found that the professional who sees the patient is a significant determinant of receipt of surveillance. Patients seen by a hepatologist or gastroenterologist were 4.5 times more likely to receive regular surveillance, and patients seen by a doctor with an academic affiliation were 2.8 times more likely to receive regular surveillance, when compared with patients seen by a primary care physician.^9^ Singal *et al* undertook a retrospective analysis of HCC cases at a large safety-net hospital in Texas, USA. They found that surveillance completion in the 2 years prior to HCC diagnosis was more likely among patients who had seen a hepatologist (OR 6.11, 95% CI 2.5 to 14.8), and less likely in patients with alcohol-related liver disease (OR 0.14, 95% CI 0.03 to 0.65). However, barriers observed in American cohorts may not be reflective of challenges faced in universal healthcare systems. The European model of state healthcare funding means that the cost of surveillance imaging is less likely to affect uptake. A review of French registry data found that low socioeconomic status was associated with failed surveillance, despite universal health coverage.^10^

HCC surveillance in the UK

The UK’s National Health Service (NHS) provides universal healthcare coverage, free at the point of use. Patients with CLD are referred by their primary care practitioner to a gastroenterology or hepatology specialist at the nearest hospital. Patients eligible for surveillance are offered biannual abdominal US. The responsibility for ongoing liver-related care and HCC surveillance lies with the specialist: imaging is requested, findings reviewed and results are communicated by the specialist clinician. All follow-up arrangements, including entry to or exit from the surveillance programme, are determined by the specialist.

District general hospitals form the backbone of acute secondary care in the UK and are local hospitals with an emergency department, general medical, surgical and maternity services. District general (secondary care) hospitals will employ consultant gastroenterologists, and occasionally consultant hepatologists, who treat a wide variety of conditions. Tertiary care hospitals, sometimes called university teaching hospitals, provide specialist and sub-specialist care: in the case of liver disease, tertiary hospitals will employ dedicated hepatologists who specialise in the treatment of liver disease. They frequently have more resources, including inpatient beds, specialist radiology and pathology services and dedicated administrative services. While the majority of liver disease is managed at secondary care, patients with rarer conditions or advanced disease may be referred to a tertiary care hospital with specialist hepatology services.

There is a paucity of data on HCC surveillance uptake in the UK. The most common route to HCC diagnosis is emergency presentation, which is associated with reduced 1-year mortality and lower likelihood of curative treatment.^11^ A 2023 UK-wide audit of surveillance practices revealed that centres self-reported an estimated 80% compliance rate of HCC surveillance.^12^ There is a clear discrepancy between these estimated figures and the 25% surveillance attendance rate reported in large, multicentre studies in Western cohorts.^13^ Regular attendance to surveillance is an independent predictor for improved survival and delineating rates of adherence is important in understanding potential barriers and facilitating strategies to overcome these.^14^ The UK has the worst outcomes of hepatobiliary cancers in high-income countries, with late-stage HCC diagnosis associated with poor quality of life, frequent hospital admissions and has marked economic impact on the health service.^15^ There is no formalised national HCC surveillance programme in any nation outside of limited countries in Asia, with surveillance being provided on an ad hoc basis, if at all.

Given the established causal link between surveillance and improved HCC outcomes, our first aim was to identify the scale of the problem to ascertain the numbers of people with CLD and cirrhosis within the community using primary care healthcare record data. We subsequently quantified surveillance uptake in a multicentre UK study with a diverse patient population, exploring sociodemographic factors that may impact on attendance rates.

## Methods

### Study population

The study was conducted in North West and South West London, which has a catchment population of 2.6 million. We conducted a review of primary care electronic records (Whole Systems Integrated Care) using Systemized Nomenclature of Medicine (SNOMED)—Clinical Terms codes to identify people with a diagnosis of CLD, chronic hepatitis or HBV. We then considered the hospitals that serve this catchment population: Imperial College London NHS Trust, London North West University Healthcare Trust (Northwick Park Hospital, Ealing Hospital and Central Middlesex Hospital) and Croydon University Hospitals Trust to identify those patients suitable for surveillance. Of these hospitals, one was a tertiary referral centre while the other four were secondary care hospitals. Eligibility for surveillance was defined by National Institue of Clinical Excellence (NICE) and European Association for the Study of the Liver EASL guidelines.^2,16^ Exclusion criteria were any patient unsuitable for HCC surveillance, including advanced decompensated liver disease, a previous or current diagnosis of HCC or liver transplantation. Eligible patients were identified by clinical nurse specialists at each hospital trust using International Classification of Diseases, 10th edition (ICD-10) codes for ‘cirrhosis’ (K70.3; K74.3; K74.6) in addition to screening gastroenterology and hepatology outpatient clinic lists manually. Where there was discrepancy or uncertainty about eligibility, records were checked by two members of the research team (MQ and VCB) and presence of cirrhosis was confirmed by radiology or histopathology records. Consensus between the two members of the research team was reached about eligibility before inclusion in the study.

Using electronic patient records, receipt of HCC surveillance (US, contrast-enhanced CT or MRI) from March 2021 to March 2023 was retrospectively collected. Cross-sectional abdominal imaging (CT or MRI) organised for other indications was included in the study.

Patients were categorised as being (1) regular attenders, (2) infrequent attenders or (3) non-attenders based on the number of imaging appointments attended over the span of 2 years. Regular attenders attended four imaging studies in 2 years; infrequent attenders attended less than four but more than zero imaging studies in 2 years and non-attenders attended no imaging studies in 2 years. Additionally, to be classed as regular attenders, the interval between any two imaging modalities was required to be <8 months. Patient follow-up finished at the first of HCC diagnosis, liver transplantation, death or 2-year date after the patient’s index visit. Figure 1 illustrates the process of data collection and analysis in a flow chart.

**Figure 1:**
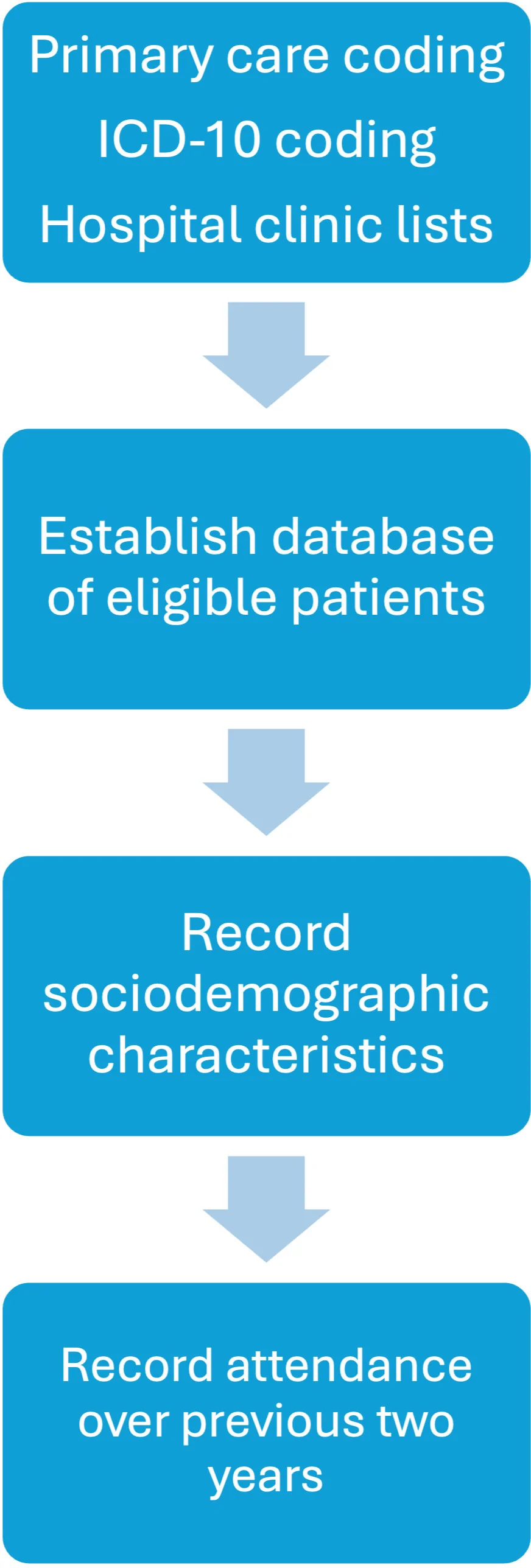
Flow chart of data collection. ICD, International Classification of Diseases, 10th edition.

#### Patient and public involvement statement

Patients were involved in developing the research question for this project. We previously hosted workshops^11^ with persons who had lived experience of CLD and cirrhosis in which they discussed their experience of HCC surveillance. They discussed barriers to attending HCC surveillance, which consequently informed our decision to investigate the impact of sociodemographic factors on surveillance uptake.

### Data collection

Sociodemographic factors including age, sex, ethnicity, aetiology of liver disease, deprivation index, hospital site and distance of home address from hospital were obtained from electronic patient records. Ethnicity was categorised according to UK Census ethnicity categories: white, black, Asian, Mixed and other.^17^ Aetiology of liver disease was classified into five categories: (1) alcohol-related liver disease, (2) HBV, (3) hepatitis C virus (HCV), (4) metabolic dysfunction-associated steatotic liver disease (MASLD) and (5) other. Index of Multiple Deprivation was determined by postcode and is used by the UK government to measure poverty at a neighbourhood level. Areas are ranked from 1 (most deprived) to 10 (least deprived). The current seven domains comprising the English Index of Deprivation are income, employment, education, health, crime, barriers to housing and services and living environment.^18^ This study was reported in concordance with Strengthening the Reporting of Observational Studies in Epidemiology (STROBE) guidelines (RECORD statement included in online supplemental appendix 1).

### Statistics

Patient data were stored on Redcap (V.13.3.3, Vanderbilt University, Tennessee, USA). Fisher exact and Mann Whitney rank-sum were used to assess associations between baseline factors and receipt of surveillance. Ethnicity was dichotomised as white versus all others, distance was dichotomised as less than or greater than 10 miles from the surveillance centre, age was grouped as <45 years, 45–70 years and >70 years and deprivation indices were collapsed to 1–5 (more deprived) and 6–10 (less deprived). Multinomial logistic regression was used to identify the impact of sociodemographic variables on surveillance attendance. Statistical analyses were performed using SPSS (IBM, V.30.0).

## Results

Electronic coding identified 2195 of people in a catchment population of 2.6 million with a diagnosis of CLD who were suitable for surveillance. This is in line with the expected population incidence of CLD of 0.08%.^19^ Following chart review, 869 patients were identified as being eligible for HCC surveillance and were under the care of hepatology, gastroenterology or infectious disease. 965 patients were not under the care of hepatology or gastroenterology. This group of patients was excluded from the final analysis as the diagnosis of CLD could not be verified from medical records. Figure 2 illustrates the number of individuals considered at each stage of the study.

**Figure 2:**
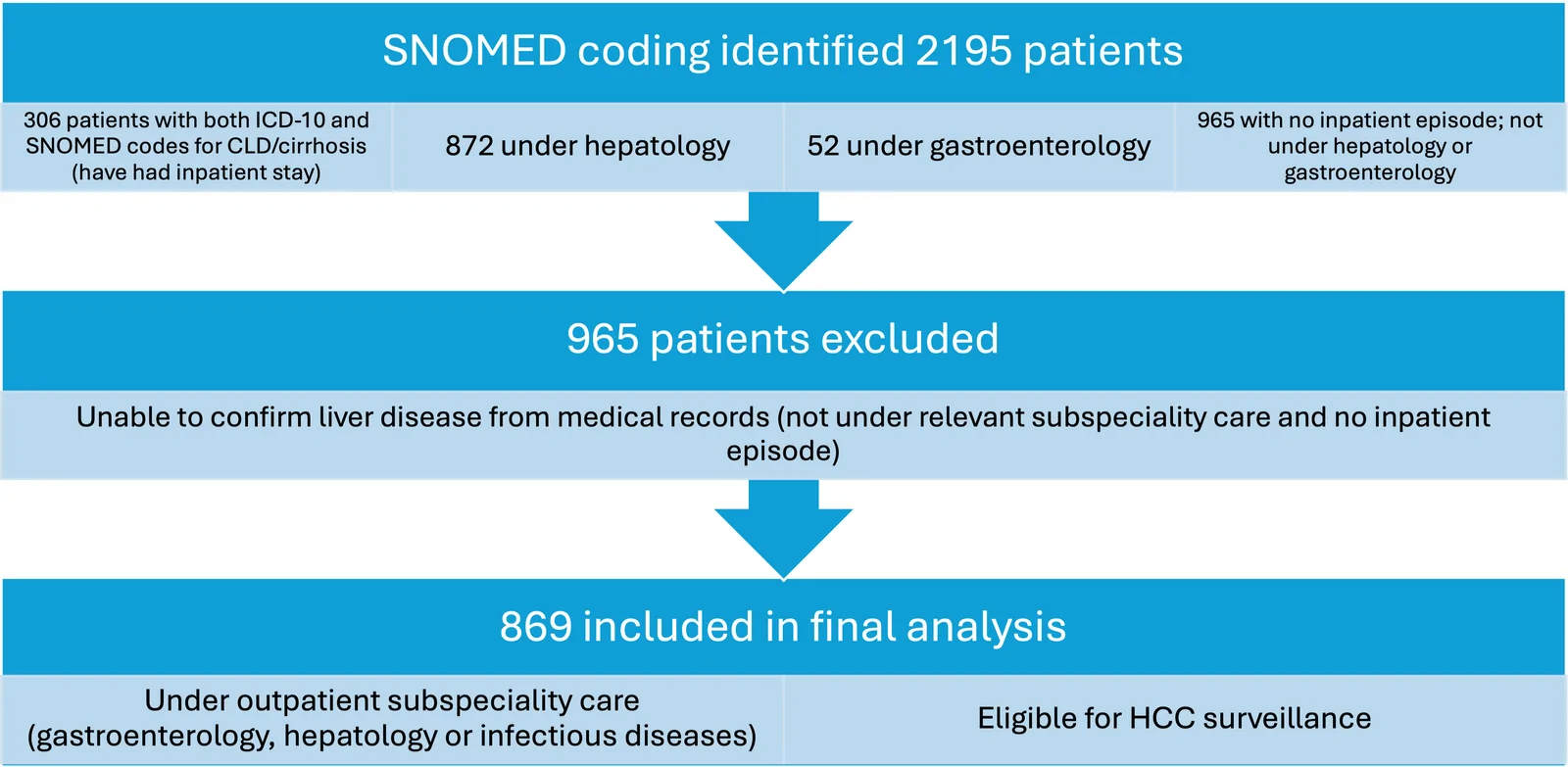
Flow chart illustrating patient inclusion in the study. CLD, chronic liver disease; HCC, hepatocellular carcinoma; SNOMED, Systemized Nomenclature of Medicine.

### Patient characteristics

Patient characteristics are detailed in table 1. The median age of the patient population was 62 years (range 23–90 years) of whom 64% were men. Most of the cohort identified as white (64%), 20% as Asian and 5% as black, with 82% living within 10 miles of the hospital which invited them to HCC surveillance. The most common cause of CLD was alcohol excess (43%) and viral hepatitis (36%: HBV 11% and HCV 25%). When considering indices of deprivation, 69% were in the bottom 50th centile. Data from participants with missing values were excluded from regression analysis.

**Table 1:** Characteristics of patients included in the study

Attendance	All patients (n=869)	Regular surveillance (%) (n=144)	Infrequent surveillance (n=635)	Non-attenders (n=90)
Age, years				
<45	87 (10%)	9 (10%)	63 (72%)	15 (17%)
45–75	673 (77%)	121 (8%)	494 (74%)	58 (18%)
>75	102 (13%)	14 (10%)	74 (73%)	14 (17%)
Missing	7 (1%)			
Sex, no. (%)				
Male	553 (64%)	87 (16%)	410 (74%)	56 (10%)
Ethnicity, no. (%)				
White	363 (42%)	21 (6%)	268 (74%)	74 (20%)
Black	40 (5%)	9 (23%)	29 (73%)	2 (5%)
Asian	172 (20%)	20 (12%)	139 (81%)	13 (8%)
Other/mixed	187 (22%)	39 (21%)	124 (66%)	24 (13%)
Missing	107 (12%)	2 (2%)	74 (69%)	31 (29%)
Aetiology of liver disease, no. (%)				
ArLD	373 (43%)	56 (15%)	287 (77%)	30 (8%)
HCV	216 (25%)	28 (13%)	142 (66%)	46 (21%)
HBV	91 (11%)	16 (17%)	67 (74%)	8 (9%)
MASLD	83 (10%)	13 (16%)	68 (82%)	2 (2%)
Other or unknown*	106 (12%)	3 (3%)	42 (40%)	13 (22%)
Socioeconomic status (Deprivation Indices), no. (%)				
1–5 (more deprived)	596 (69%)	97 (16%)	430 (72%)	69 (12%)
6–10 (less deprived)	254 (29%)	44 (17%)	196 (77%)	14 (6%)
Unknown	19 (2%)			
Distance from hospital, no. (%)				
≤10 miles	715 (82%)	76 (11%)	528 (74%)	111 (15%)
>10 miles	114 (13%)	8 (7%)	76 (67%)	30 (26%)
Unknown	40 (5%)			
Hospital type				
Tertiary	379 (44%)	124 (33%)	225 (59%)	30 (8%)
Secondary	463 (53%)	20 (4%)	383 (83%)	60 (13%)
Unknown	27 (3%)			

* ’Other or unknown’ liver disease refers to patients with autoimmune liver disease (including primary biliary cholangitis and primary sclerosing cholangitis), haemochromatosis or cryptogenic cirrhosis

ArLD, alcohol-related liver disease; HBV, hepatitis B virus; HCV, hepatitis C virus; MASLD, metabolic dysfunction-associated steatotic liver disease .

### Determinants of HCC surveillance

Surveillance was performed at least once in 779 (90%) patients over the 2 year period. Of the study population, only 17% (n=144) attended surveillance appointments regularly; the majority were infrequent attenders (73%) and 10% did not attend for any surveillance appointments. Of the patients classified as infrequent attenders; 193 (30%) attended for one scan, 224 (35%) completed two surveillance scans and 166 (26%) had three scans during the 2-year period; of note, 52 patients (8%) presented four times but outside of the 6–8 month screening interval. One secondary care centre had no patients that had attended surveillance regularly.

We explored potential sociodemographic factors associated with regular attendance.^13^ First, multinomial logistic regression was undertaken with regular surveillance as the reference category. When considering non-attendance, the strongest predictor for regular attendance was surveillance organised by a tertiary referral centre compared with those undergoing surveillance at secondary care hospitals (OR 0.08, 95% CI 0.04 to 0.2, p<0.001). Similarly, those less deprived were more likely to be regular attenders (OR 0.4, 95% CI 0.2 to 0.8, p=0.008). Those aged between 45–75 years were more likely to be non-attenders compared with the older and younger cohorts (OR 2.7, 95% CI 1.2 to 6.0, p=0.014). When considering infrequent attendance in comparison to regular attendance, those less deprived were more likely to be regular attenders (OR 0.4, 95% CI 0.2 to 1.0, p=0.006). Conversely, those aged between 45–75 years were less likely to be regular attenders compared with the older and younger cohorts (OR 2.5, 95% CI 1.3 to 4.5, p=0.004) (table 2).

**Table 2:** Multinomial regression analysis of factors influencing regular surveillance attendance

	OR	95% CI	P value
Infrequent			
Age, years			
<45	0.9	0.4 to 2.1	0.9
45–75	2.5	1.3 to 4.5	**0.004**
>75	ref	ref	ref
Socioeconomic status			
Deprivation index (more deprived)	0.4	0.2 to 1.0	**0.006**
Deprivation index (less deprived)	ref	ref	ref
Hospital type			
Secondary	1.0	0.6 to 1.6	0.9
Tertiary	ref	ref	ref
Non-attender			
Age, years			
<45	1.09	0.3 to 3.5	0.9
45–75	2.7	1.2 to 6.0	**0.014**
>75	ref	ref	ref
Socioeconomic status			
Deprivation index (more deprived)	0.4	0.2 to 0.8	**0.008**
Deprivation index (less deprived)	ref	ref	ref
Hospital type			
Secondary	0.08	0.04 to 0.2	**<0.001**
Tertiary	ref	ref	ref

Bold are those that are p<0.05.

Ref, reference group.

Given the large cohort of patients undergoing infrequent surveillance, we further considered sociodemographic predictors of infrequent attendance compared with non-attenders. The strongest, independent predictor for non-attendance was surveillance organised by a secondary hospital compared with those undergoing surveillance at a tertiary referral centre (OR 11.4, 95% CI 6.6 to 19.3, p<0.001).

## Discussion

Biannual surveillance for HCC for patients with cirrhosis is recommended by all international professional bodies, with adherence associated with early diagnosis and improved survival outcomes.^20,21^ Our study suggests that these guidelines are not implemented in clinical practice. We report low rates of regular surveillance with only 17% of patients undergoing regular, biannual surveillance over a 2-year period. However, contrary to previous work, we observed that most patients presented to surveillance infrequently with 73% of patients attending at least one surveillance scan in a 2-year period. We explored socioeconomic determinants of HCC surveillance uptake and identified surveillance organised by district general hospitals, deprivation and age as strong determinants of reduced attendance to surveillance appointments. To our knowledge, this is the largest European cohort study to investigate sociodemographic determinants of surveillance attendance in a population reflective of the diversity of liver disease, representative of most social healthcare systems.

Using primary care coding, we identified 2195 of people in a catchment population of 2.6 million with a diagnosis of CLD. This is in line with the expected population incidence of CLD of 0.08%.^19^ However, only 869 patients were identified as suitable for HCC surveillance and receiving outpatient care for their liver disease. This may be due to incorrect coding both within primary care and within the hospital setting, or that primary care coding captures patients who are no longer suitable for surveillance who have been subsequently discharged back to the community for ongoing care. However, given the large discrepancy in numbers it is possible that at-risk individuals with CLD may not have been referred from primary care for HCC surveillance. This is supported by work by Wong and colleagues that suggested that there was poor knowledge of CLD and surveillance in primary care which directly impacts on attendance.^22^ Work by ourselves has shown that patients have high levels of trust in their primary care physician with their endorsement being a central motivator of attendance.^23^ As the UK moves to institute a national HCC surveillance programme, understanding of primary care knowledge and attitudes towards surveillance will be key in ensuring regular attendance.^16^

Overall, attendance for HCC surveillance was poor: only 17% of patients were regular attenders over the 2 year study period. This supports Burton and colleagues’ 2024 review of the route to diagnosis of HCC in the UK, which found that only 17% of HCC patients were referred via the outpatient route, suggesting that only 17% of cases were diagnosed via surveillance. Concerningly, the most common route to diagnosis was via emergency presentation.^11^ We found one secondary care centre had no patients that had attended surveillance regularly. This reflects the lack of cohesive national policy on HCC surveillance: some centres (including the one studied) have a ‘one strike’ policy in which patients who do not attend for surveillance are no longer invited for further scans.

Our findings for regular attendance are in keeping with meta-analysis of studies conducted in the USA demonstrating pooled regular attendance of <20%.^24^ However, the meta-analysis and subsequent studies report much higher levels of non-attendance.^25,26^ Most of our cohort were infrequent attenders, presenting at least once during the 2 year period. The reasons for infrequent attendance have not been previously explored. Studies have identified system and clinician level barriers such as timely booking of scans, communication with patients and radiology capacity as potential obstacles in surveillance attendance.^23^ We found that care under a secondary care hospital was the strongest predictor of both infrequent and non-attendance. While all these hospital sites have hepatology clinics, these are not transplant or hepatology specialist care centres and do not have dedicated CNS: this finding supports previous work in the USA demonstrating that HCC surveillance receipt was higher for patients under tertiary or subspecialty hepatology care.^27^ This may be due to the dedicated time and resources available at specialist hepatology units compared with busy general hospitals with lengthy waiting lists and fewer dedicated staff. The role of specialist CNS and patient navigation have been shown in other cancers and in HCC to improve attendance through the provision of education and support particularly to marginalised communities.^28^ Of concern, UK clinicians believe surveillance uptake is good: clinicians self-reported surveillance uptake of 80% in a recent review of UK hepatology practice, despite acknowledging that most centres do not audit surveillance uptake.^12^ The marked discrepancy between self-reported and real figures highlights the need for physician education regarding surveillance as well as the requirement for regular audit of surveillance practices.

Patients of a lower socioeconomic status were more likely to be non-attenders at surveillance. This is in keeping with Singal and colleagues' work on HCC surveillance in the USA, and broader uptake of cancer screening in the UK and internationally.^29,30^ Lack of education, health illiteracy, difficulties scheduling time off work, and affordability of transport to and from appointments may all contribute to poor attendance at screening. Existing research demonstrates that patients of lower socioeconomic status with liver disease face an increased risk of all-cause mortality: it is key therefore to target these patients when designing interventions to improve screening uptake.^31^ Similarly, previous work has reported that patients aged between 51 years and 64 years were more likely to attend for surveillance inconsistently compared with both older and younger age groups.^26^ This is consistent with our work and may relate to competing lifestyle issues in this group including work, transport and family challenges which should be explored in future work.

North West and South London are areas of high culturally and linguistically diverse (CALD) communities. However, no difference in attendance rates between ethnic groups when adjusted for other sociodemographic variables was observed. This is in contrast with previous work in bowel, breast and cervical cancer screening in the UK whereby CALD communities are less likely to engage in screening programmes.^32,33^ Similarly, results from a meta-analysis of HCC surveillance suggest that people who identify as White are more likely to attend appointments.^24^ A large cohort study from the UK of 8.52 million individuals suggested that Asian and black people are more likely to develop HCC.^34^ However, no differences were noted in the cancer stage of diagnosis, supporting our findings that ethnicity per se does not impact on attendance.

Attendance at HCC surveillance is poor when examined in the context of breast (65%), cervical and bowel cancer screening (57–66%) uptake within the UK.^35,36^ The reasons for this require in-depth examination into the barriers to HCC surveillance. We postulate that this is due to several factors: liver disease disproportionately affects marginalised communities who are less likely to engage with healthcare and will therefore have lower attendance at surveillance appointments.^37^ This group of patients express high levels of stigma which limit engagement with healthcare systems.^23^ There have been several successful media campaigns to raise awareness and encourage attendance at breast, cervical and bowel cancer screening which has not been the case with HCC surveillance. Additionally, breast, cervical and bowel cancer screening programmes are true ‘screening’ programmes eligible to the whole population and may benefit from peer and family encouragement; this contrasts with HCC surveillance which targets high-risk individuals who may be unaware of their cancer risk. Recent work by our group suggests that at-risk individuals are often not aware of the reason for liver cancer surveillance, leading to de-prioritisation of appointment attendance.^23^ This supports a large American study which found that patient knowledge deficits (for example, believing surveillance was unnecessary in the absence of symptoms) were associated with suboptimal surveillance attendance.^29^

There are some limitations to our study. Due to a lack of comprehensive HCC surveillance database generated through ICD-10 coding, not all eligible patients will have been included in our analysis. It is possible that the true figures for attendance are even lower, as those patients ‘missed’ by clinicians during identification are more likely to have been missed from follow-up and imaging requests. For example, our analysis of SNOMED data found that 965 patients were coded as having CLD in primary care but were not under the care of a gastroenterologist or hepatologist. The reasons for this are unclear and should be the focus of further work. Notably, it was not possible to confirm the reasons for non-receipt of a surveillance episode on electronic patient records, so we are unable to understand if suboptimal receipt was due to (1) surveillance not being requested by the clinician or (2) patient did not attend for surveillance appointment. We hypothesise that it is due to a combination of both factors, and influenced by the ‘one strike’ policy previously discussed: a patient not attending their appointment would not have further appointments booked for them by the clinician.

The study period March 2021–March 2023 includes the COVID pandemic and post-pandemic recovery phase. NHS data show that there was widespread disruption to outpatient bookings and attendance until March 2022. Therefore, our figures on attendance might underestimate overall trends. However, it is important to recognise the direct and indirect effects of the pandemic on HCC surveillance to begin to mitigate the disruptive effects of this period. Further work should examine surveillance attendance in this population in the post-pandemic era. Finally, all hospitals included in this study were in the Greater London area: wider trends across the UK have not been assessed and would be a valuable focus for further work. However, the diversity of patients included in this study ensures that there is representation across aetiologies, ethnicities and socio-economic groups. One caveat to this is that MASLD was likely underreported in our cohort, with only 10% of patients identified as MASLD. This might be due to incomplete diagnosis or coding in those with multiple aetiologies, for example ArLD/MASLD or HBV/MASLD.

This research has highlighted considerable scope for service improvement and future study in HCC surveillance uptake. The creation of a national surveillance database will be of critical importance in ensuring that surveillance is offered to all eligible patients and will enable individual trusts to record and audit attendance. Future work will involve in-depth examination of patient-reported and physician-reported barriers to surveillance attendance via semistructured interviews. This will highlight areas where intervention is necessary, for example, transportation or educational barriers, or addressing structural challenges such as radiology capacity.

In conclusion, we present the first UK study exploring sociodemographic determinants of HCC surveillance uptake. Regular surveillance uptake is low, and concerningly, much lower than referring clinicians recognise. Given the poor outcomes for HCC, there is an urgent need to improve surveillance uptake across all groups—especially in patients from a lower socioeconomic background and those under the care of non-specialist hospitals. Our findings highlight the need for improvement in care for patients at risk of HCC, and further research identifying interventions to optimise HCC surveillance uptake.

## Supplementary material

10.1136/bmjph-2026-005067 online supplemental file 1

## Data Availability

Data are available upon reasonable request.

## References

[1] Ferlay J, Colombet M, Soerjomataram I (2019). Estimating the global cancer incidence and mortality in 2018: GLOBOCAN sources and methods. Int J Cancer.

[2] European Association for the Study of the Liver. Electronic address eee, European Association for the Study of the L (2018). EASL Clinical Practice Guidelines: Management of hepatocellular carcinoma. J Hepatol.

[3] Heimbach JK, Kulik LM, Finn RS (2018). AASLD guidelines for the treatment of hepatocellular carcinoma. Hepatology.

[4] Zhang BH, Yang BH, Tang ZY (2004). Randomized controlled trial of screening for hepatocellular carcinoma. J Cancer Res Clin Oncol.

[5] Singal AG, Zhang E, Narasimman M (2022). HCC surveillance improves early detection, curative treatment receipt, and survival in patients with cirrhosis: A meta-analysis. J Hepatol.

[6] Cucchetti A, Cescon M, Erroi V (2013). Cost-effectiveness of liver cancer screening. Best Pract Res Clin Gastroenterol.

[7] Wolf E, Rich NE, Marrero JA (2021). Use of Hepatocellular Carcinoma Surveillance in Patients With Cirrhosis: A Systematic Review and Meta-Analysis. Hepatology.

[8] Singal AG, Yopp AC, Gupta S (2012). Failure rates in the hepatocellular carcinoma surveillance process. Cancer Prev Res (Phila).

[9] Davila JA, Morgan RO, Richardson PA (2010). Use of surveillance for hepatocellular carcinoma among patients with cirrhosis in the United States. Hepatology.

[10] Caillet P, Balusson F, Ganne-Carié N (2025). Three-year of hepatocellular carcinoma surveillance in patients with cirrhosis diagnosed between 2009 and 2013: a cohort study based on the French National Health Data System (SNDS) claims data. Front Oncol.

[11] Burton A, Wilburn J, Driver RJ (2024). Routes to diagnosis for hepatocellular carcinoma patients: predictors and associations with treatment and mortality. Br J Cancer.

[12] Scott RA, Cross TJS, Clarke C (2023). Outcomes of National Survey of the Practice of Hepatocellular Carcinoma Surveillance. J Hepatocell Carcinoma.

[13] Farvardin S, Patel J, Khambaty M (2017). Patient-reported barriers are associated with lower hepatocellular carcinoma surveillance rates in patients with cirrhosis. Hepatology.

[14] Costentin CE, Layese R, Bourcier V (2018). Compliance With Hepatocellular Carcinoma Surveillance Guidelines Associated With Increased Lead-Time Adjusted Survival of Patients With Compensated Viral Cirrhosis: A Multi-Center Cohort Study. Gastroenterology.

[15] Arnold M, Rutherford MJ, Bardot A (2019). Progress in cancer survival, mortality, and incidence in seven high-income countries 1995-2014 (ICBP SURVMARK-2): a population-based study. Lancet Oncol.

[16] England N (2024). Hepatocellular carcinoma surveillance: minimum standards 2024. https://www.england.nhs.uk/long-read/hepatocellular-carcinoma-surveillance-minimum-standards.

[17] Statistics OfN (2026). Ethnic group, England and Wales: Census 2021 2022. https://www.ons.gov.uk/peoplepopulationandcommunity/culturalidentity/ethnicity/bulletins/ethnicgroupenglandandwales/census2021.

[18] Gov.uk (2019). English indices of deprivation 2019. https://www.gov.uk/government/statistics/english-indices-of-deprivation-2019.

[19] Williams R, Alexander G, Aspinall R (2018). Gathering momentum for the way ahead: fifth report of the Lancet Standing Commission on Liver Disease in the UK. Lancet.

[20] European Association for the Study of the Liver. Electronic address eee, Clinical Practice Guideline P, Chair, representative EGB, Panel m (2021). EASL Clinical Practice Guidelines on non-invasive tests for evaluation of liver disease severity and prognosis – 2021 update. J Hepatol.

[21] NICE (2023). Quality statement 4: surveillance for hepatocellular carcinoma. https://www.nice.org.uk/guidance/qs152/chapter/quality-statement-4-surveillance-for-hepatocellular-carcinoma.

[22] Wong RJ, Jones PD, Niu B (2024). Clinician-Level Knowledge and Barriers to Hepatocellular Carcinoma Surveillance. *JAMA Netw Open*.

[23] Qurashi M, von Wagner C, Sharma R (2024). Optimising Surveillance in Hepatocellular Carcinoma: Patient-Defined Obstacles and Solutions. J Hepatocell Carcinoma.

[24] Singal AG, Yopp A, S Skinner C (2012). Utilization of hepatocellular carcinoma surveillance among American patients: a systematic review. J Gen Intern Med.

[25] Singal AG, Narasimman M, Daher D (2024). Effectiveness of mailed outreach and patient navigation to promote HCC screening process completion: a multicentre pragmatic randomised clinical trial. Gut.

[26] Davila JA, Henderson L, Kramer JR (2011). Utilization of surveillance for hepatocellular carcinoma among hepatitis C virus-infected veterans in the United States. Ann Intern Med.

[27] Ahmed Mohammed HA, Yang JD, Giama NH (2017). Factors Influencing Surveillance for Hepatocellular Carcinoma in Patients with Liver Cirrhosis. Liver Cancer.

[28] Mosquera I, Todd A, Balaj M (2023). Components and effectiveness of patient navigation programmes to increase participation to breast, cervical and colorectal cancer screening: A systematic review. Cancer Med.

[29] Singal AG, Tiro JA, Murphy CC (2021). Patient-Reported Barriers Are Associated With Receipt of Hepatocellular Carcinoma Surveillance in a Multicenter Cohort of Patients With Cirrhosis. Clin Gastroenterol Hepatol.

[30] de Klerk CM, Gupta S, Dekker E (2018). Expert Working Group “Coalition to reduce inequities in colorectal cancer screening” of the World Endoscopy O. Socioeconomic and ethnic inequities within organised colorectal cancer screening programmes worldwide. Gut.

[31] Ayyala-Somayajula D, Dodge JL, Farias A (2023). Healthcare affordability and effects on mortality among adults with liver disease from 2004 to 2018 in the United States. J Hepatol.

[32] Bae J, Awammi M, Macnair A (2022). Cervical smear uptake in Black, Asian and minority ethnic and learning disability populations. Clin Med (Lond).

[33] Szczepura A, Price C, Gumber A (2008). Breast and bowel cancer screening uptake patterns over 15 years for UK south Asian ethnic minority populations, corrected for differences in socio-demographic characteristics. BMC Public Health.

[34] Liao W, Coupland CAC, Innes H (2023). Disparities in care and outcomes for primary liver cancer in England during 2008-2018: a cohort study of 8.52 million primary care population using the QResearch database. EClinicalMedicine.

[35] Taylor-Phillips S, O’Sullivan E, Kearins O (2013). The effects of a UK review of Breast Cancer Screening on Uptake: an observational before/after study. J Med Screen.

[36] Lo SH, Halloran S, Snowball J (2015). Colorectal cancer screening uptake over three biennial invitation rounds in the English bowel cancer screening programme. Gut.

[37] UK CR (2023). Liver cancer statistics. https://www.cancerresearchuk.org/health-professional/cancer-statistics/statistics-by-cancer-type/liver-cancer#heading-Zero.

